# Etiology and prevention of prevalent types of cancer

**DOI:** 10.29245/2572-9411/2017/3.1093

**Published:** 2017-05-02

**Authors:** Ercole L. Cavalieri, Eleanor G. Rogan

**Affiliations:** 1 Eppley Institute for Research in Cancer and Allied Diseases, University of Nebraska Medical Center, Omaha, NE 68198-6805, USA; 2 Department of Environmental, Agricultural and Occupational Health, College of Public Health, University of Nebraska Medical Center, Omaha, NE 68198-4388, USA

**Keywords:** Biomarkers of cancer risk, Cancer initiation, Cancer prevention, Catechol estrogen-3,4-quinones, Depurinating estrogen-DNA adducts, Estrogen metabolism

## Abstract

Endogenous estrogens become carcinogens when excessive catechol estrogen quinone metabolites are formed. Specifically, the catechol estrogen-3,4-quinones can react with DNA to produce a large amount of specific depurinating estrogen-DNA adducts, formed at the N-3 of Ade and N-7 of Gua. Loss of these adducts leaves apurinic sites in the DNA, which can generate subsequent cancer-initiating mutations. Unbalanced estrogen metabolism yields excessive catechol estrogen-3,4-quinones, increasing formation of the depurinating estrogen-DNA adducts and the risk of initiating cancer. Evidence for this mechanism of cancer initiation comes from studies in vitro, in cell culture, in animal models and in human subjects. High levels of estrogen-DNA adducts have been observed in women with breast, ovarian or thyroid cancer, and in men with prostate cancer or non-Hodgkin lymphoma. Observation of high levels of depurinating estrogen-DNA adducts in high risk women before the presence of breast cancer indicates that adduct formation is a critical factor in breast cancer initiation. Two dietary supplements, *N*-acetylcysteine and resveratrol, complement each other in reducing formation of catechol estrogen-3,4-quinones and inhibiting formation of estrogen-DNA adducts in cultured human and mouse breast epithelial cells. They also inhibit malignant transformation of these epithelial cells. In addition, formation of adducts was reduced in women who followed a Healthy Breast Protocol that includes *N*-acetylcysteine and resveratrol. Blocking initiation of cancer prevents promotion, progression and development of the disease. These results suggest that reducing formation of depurinating estrogen-DNA adducts can reduce the risk of developing a variety of types of human cancer.

Cancer is often a problem of chemical carcinogenesis. This means that chemicals are frequently involved in the process leading to cancer. The chemicals that cause much of human cancer are the estrogens, which can form excessive carcinogenic catechol estrogen-3,4-quinone metabolites (Figure 1).

## Estrogen metabolism leading to the formation of estrogen-DNA adducts

**Figure 1:**
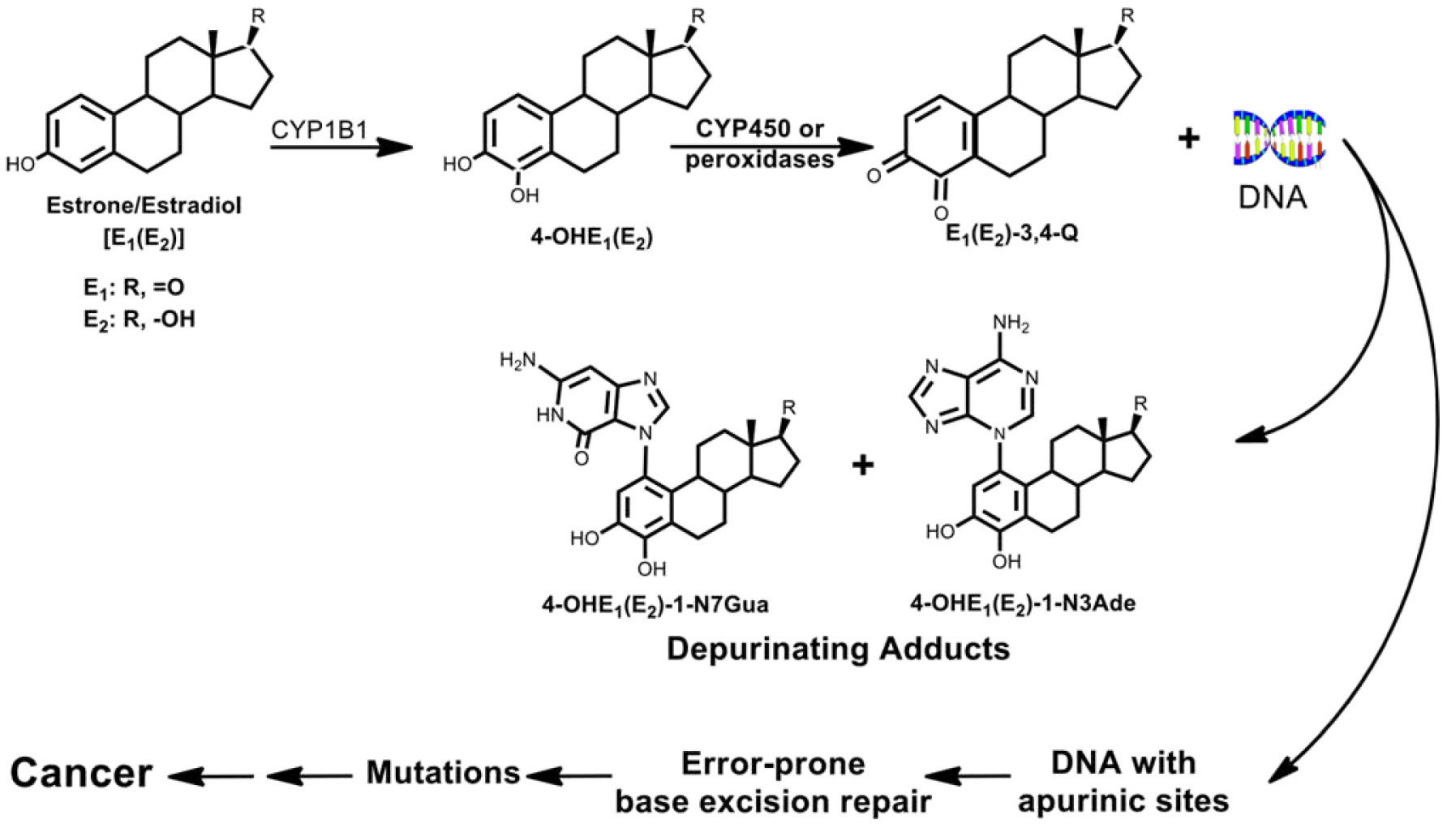
Major metabolic pathway (97%) in cancer initiation by estrogens.

**Figure 2:**
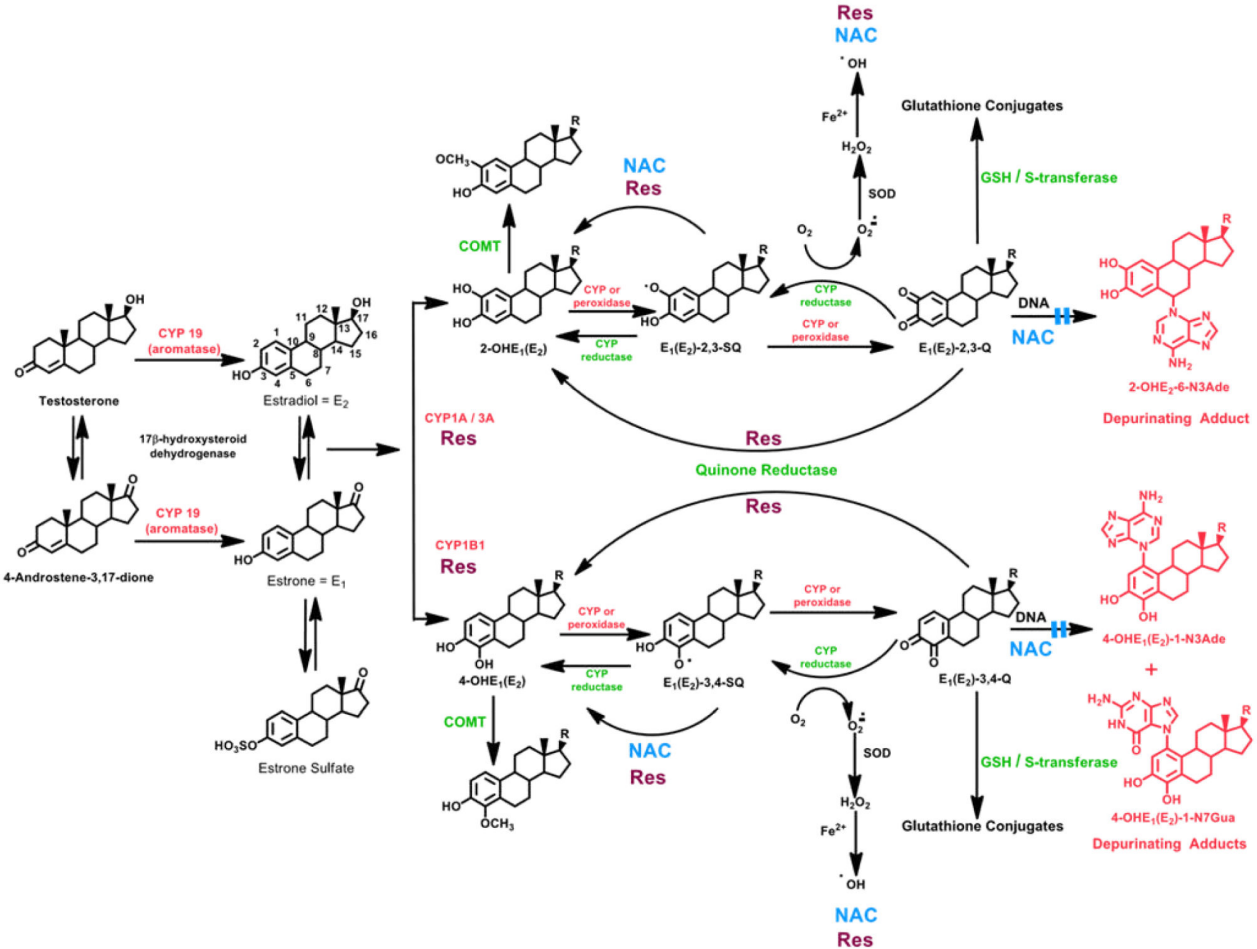
Formation of estrogens, catechol estrogen metabolic pathway of estrogens and depurinating DNA adducts of estrogens. Activating enzymes and depurinating DNA adducts are in red, and protective enzymes are in green. N-Acetylcysteine (NAC, shown in blue) and resveratrol (Res, shown in burgundy) indicate various steps where NAC and Res can ameliorate unbalanced estrogen metabolism and reduce formation of depurinating estrogen-DNA adducts.

Estrogens are metabolized via two major pathways: formation of 16α-hydroxyestrone (estradiol) [E_1_(E_2_)] (not shown in Figure 2) and formation of the catechol estrogens 2-OHE_1_(E_2_) and 4-OHE_1_ (E_2_)^1^. Cytochrome P450 (CYP) 1A1 hydroxylates E_1_ and E_2_ preferentially at the 2-position, whereas CYP1B1 hydroxylates almost exclusively at the 4-position^2-4^, and the 4-OHE_1_(E_2_) are the most important metabolites in cancer initiation (Figure 1)^5-7^. The most common pathway of conjugation of catechol estrogens in extrahepatic tissues is *O*-methylation, catalyzed by catechol-*O*-methyltransferase (COMT)^8,9^. When COMT activity is low, competitive oxidation of catechol estrogens to semiquinones and then to quinones, catalyzed by CYP or peroxidases, can occur (Figure 2).

Following formation of catechol estrogen quinones, they can be inactivated by reaction with glutathione (GSH) or reduction to their catechols by quinone reductase (NQO1)^10,11^, a protective enzyme induced by various compounds^12^. If the catechol estrogen quinones are not eliminated by protective processes, they can react with DNA (Figure 2). Catechol estrogen quinones covalently bind to DNA to form two types of adducts: stable ones that remain in DNA unless removed by repair and depurinating adducts that are lost from DNA by destabilization of the glycosyl bond^13,14^.

## Apurinic sites and mutations

Evidence that depurinating estrogen-DNA adducts play a critical role in cancer initiation comes from a correlation between depurinating estrogen-DNA adducts that generate apurinic sites and oncogenic Harvey (H)-*ras* mutations in preneoplastic mouse skin^15^ and rat mammary gland^16^. Apurinic sites occur spontaneously in cells^17^. In mouse skin treated with E_2_-3,4-quinone (Q), however, the number of apurinic sites is 145 times greater than the number of spontaneously formed sites^15,18^, presumably overwhelming the repair mechanism and generating mutations.

Estrogens have been thought to be epigenetic carcinogens that stimulate abnormal cell proliferation through estrogen receptor (ER)-mediated processes^19-21^. This stimulated cell proliferation could lead to increased genetic damage and initiate cancer^20-22^. We do not consider ER-mediated processes to be significantly involved in cancer initiation for a variety of reasons. First, 4-OHE_1_(E_2_) have higher carcinogenic potency than 2-0 HE_1_(E_2_)^5-7^, which cannot be explained by ER-mediated processes. Second, ERKO/Wnt-1 mice, which have no functional ER-α, develop estrogen-induced mammary tumors^23-25^.

When mouse skin treated with E_2_-3,4-Q was analyzed for both formation of depurinating estrogen-DNA adducts and H-*ras* mutations, predominantly the depurinating 4-OHE_1_(E_2_)-1-N3Ade and 4-OHE_1_(E_2_)-1-N7Gua adducts were formed (>99%) and mostly A to G mutations were detected only 6-12 h after treatment^15^. Similar results were obtained when rat mammary gland was treated with E_2_-3,4-Q^16^.

Estrogen mutagenicity has also been demonstrated in transfected Big Blue^®^ rat2 embyronic cells^26^ and Big Blue^®^ rats treated with 4-OHE_2_^18^. The generation of mutations in mouse skin, rat mammary gland and cultured cells shows that estrogens are, indeed, directly mutagenic.

## Cancer initiation

Imbalanced estrogen metabolism can lead to excessive production of catechol estrogen-3,4-quinones that generate estrogen-DNA adducts. These imbalances can lead to excessive formation of estrogens because of overexpression of CYP19 (aromatase)^27-29^ and unregulated sulfatase that converts stored E_1_-sulfate into E_1_^30,31^. If CYP1B1 is overexpressed, higher levels of 4-OHE_1_(E_2_) will be available^2-4^ for conversion into E_1_(E_2_)-3,4-Q, the strongest carcinogenic metabolites of estrogens (Figure 1). Polymorphic variations in COMT can limit the activity of this enzyme, allowing more 4-OHE_1_(E_2_) to be converted into E_1_(E_2_)-3,4-Q^9,32^. Polymorphisms in NQ01 can lead to decreased reduction of the catechol estrogen quinones back to catechol estrogens^33^, again leaving more quinones available to react with DNA, unless they are removed by reaction with GSH.

Imbalances in estrogen metabolism have been observed in several animal models for estrogen carcinogenicity: the kidney of male Syrian golden hamsters^34^, prostate of Noble rats^35^ and mammary gland of transgenic estrogen receptor-α knock-out mice^24^. These imbalances have also been observed in breast tissue of women with breast cancer. In tumor-adjacent breast tissue, the levels of 4-OHE_1_(E_2_) were almost four-times higher than those in breast tissue from women without breast cancer^36^. The breast tissue from women with breast cancer also demonstrated greater expression of the estrogen-activating enzymes CYP19 and CYP1B1, compared to women without breast cancer, who exhibited greater expression of the estrogen-protective enzymes COMT and NQO1^37^.

The ability of estrogens to induce malignant transformation of mammalian cells has been demonstrated in cultured human and mouse mammary epithelial cells. When the human non-transformed MCF-10F cells were treated with E_2_, depurinating estrogen-DNA adducts were formed and the cells were malignantly transformed in a dose-dependent manner^38^. Similarly, when non-transformed mouse E6 cells were treated with 4-OHE_2_ or E_2_-3,4-Q, the cells formed depurinating estrogen-DNA adducts and were malignantly transformed in a dose-dependent manner^39^. Such studies demonstrate a critical role of depurinating estrogen-DNA adducts in the processes leading to malignant transformation.

## Depurinating estrogen-DNA adducts: biomarkers of cancer risk and initiation

The first evidence that depurinating estrogen-DNA adducts play a major role in cancer initiation was obtained from a correlation between the sites of formation of depurinating estrogen-DNA adducts and H-*ras* mutations in mouse skin and rat mammary gland treated with the ultimate carcinogenic metabolite E_2_-3,4-Q^15,16^. Estrogen metabolites, estrogen-GSH conjugates and depurinating estrogen-DNA adducts can now be analyzed in human serum and urine by using ultraperformance liquid chromatography/tandem mass spectrometry (UPLC-MS/MS), The ratio of the depurinating adducts, 4-OHE_1_(E_2_)-1-N3Ade, 4-OHE_1_(E_2_)-1-N7Gua and 2-OHE_1_(E_2_)-6-N3Ade, to estrogen metabolites and conjugates provides a reliable measure of the balance or imbalance of estrogen metabolism in a person:

ratio=(4−OHE1(E2)−1−N3Ade+4−OHE1(E2)−1−N7Gua4−catechol estrogens+4−catechol estrogen conjugates+)(2−OHE1(E2)−6−N3Ade2−catechol estrogens+2−catechol estrogen conjugates)×1000

This ratio serves as a biomarker for risk of developing estrogen-initiated cancer^40,41^.

**Figure 3:**
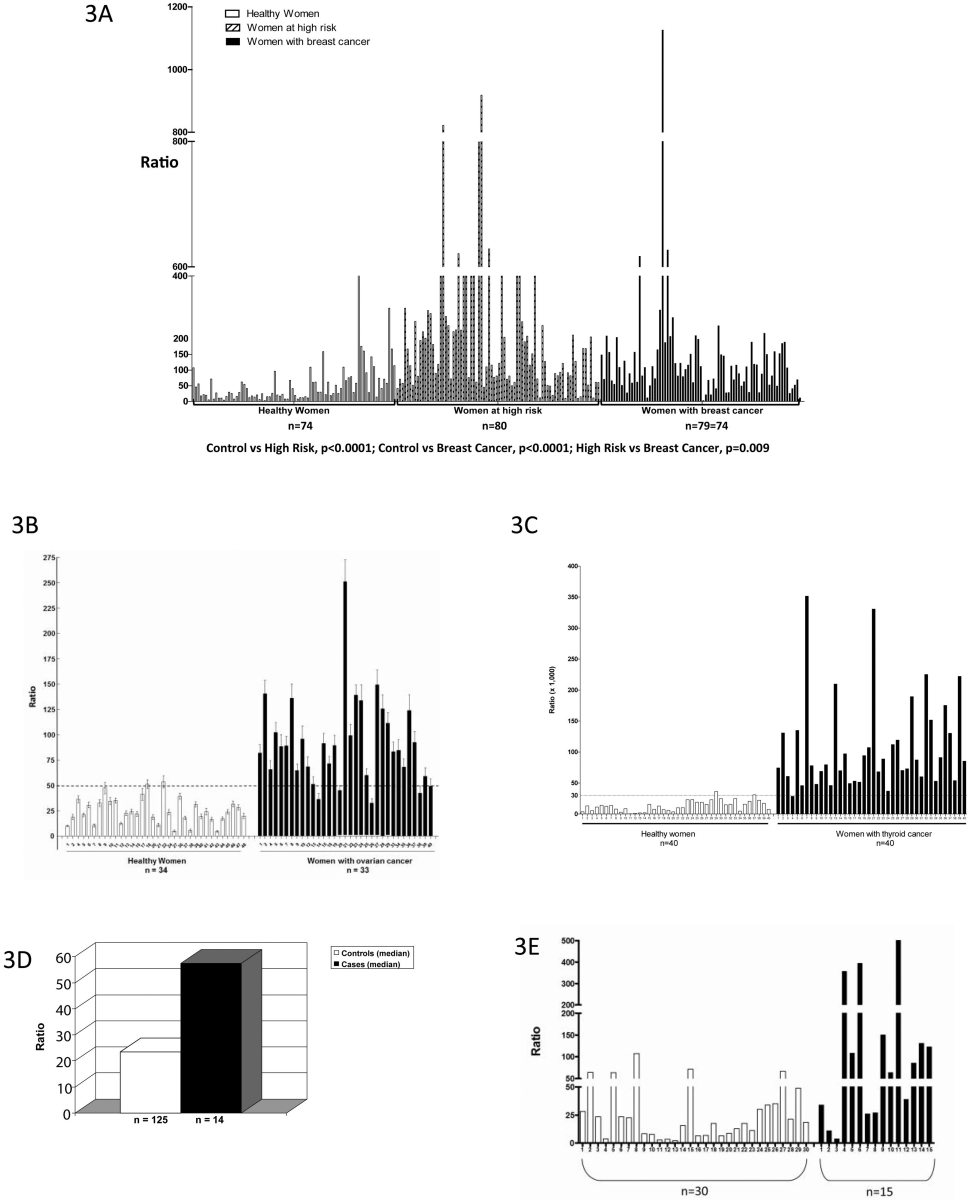
Ratios of depurinating estrogen-DNA adducts to estrogen metabolites and estrogen conjugates in (*a*) serum samples from healthy women, high-risk women and women with breast cancer^43^; (*b*) urine samples from women with and without ovarian cancer (p<0.0001)^44^; (*c*) urine samples from women with and without thyroid cancer (p<0.0001). The dotted line at a ratio of 50 is the cut-point for sensitivity and specificity of the ratio^45^; (*d*) urine samples from men with and without prostate cancer (mean levels, p<0.001)47; and (*e*) urine samples from men with and without NHL (p<0.007)^48^.

Caucasian women diagnosed with breast cancer, or at normal or high risk for developing the disease, have been investigated in three case-control studies^40,42,43^. In the first two, a spot urine sample was analyzed by UPLC-MS/MS and the estrogen-DNA adduct ratio (see above) was calculated for each subject^40,42^. The ratios in the high-risk women and those diagnosed with breast cancer were significantly higher than those in the normal-risk women (p<0.001 in both studies)^40,42^. The third study used serum samples, and similar results were obtained, with even greater differences between the normal-risk women and high-risk women or those with breast cancer [p<0.0001, Figure 3(*a*)]^43^. No differences in the results were observed when the subjects were separated into pre- and peri/postmenopausal groups^43^. These results, especially the high ratios observed in high-risk women, indicate that formation of estrogen-DNA adducts plays a critical role in the etiology of breast cancer.

The ratio of estrogen-DNA adducts to metabolites and conjugates was also investigated in women with and without ovarian cancer^44^. The women diagnosed with ovarian cancer demonstrated higher ratios than the controls [p<0.0001, Figure 3 (*b*)]. DNA from saliva samples was purified and single nucleotide polymorphisms (SNPs) were analyzed in the genes for the estrogen-activating enzyme CYP1B1 (V432L) and the protective enzyme COMT (V158M)^44^. The women with two copies of both the low-activity COMT allele plus the high-activity CYP1B1 allele demonstrated much higher values of the DNA adduct ratio, and the odds ratio for ovarian cancer was 6-fold higher compared to women with the normal-activity alleles of the enzymes. These combined results suggest that initiation of ovarian cancer is strongly associated with unbalanced estrogen metabolism leading to formation of estrogen-DNA adducts.

When estrogen metabolites, conjugates and depurinating DNA adducts were analyzed in a small study of urine samples from women with and without thyroid cancer, the women with thyroid cancer had much higher ratios of estrogen-DNA adducts to estrogen metabolites and conjugates [p<0.0001, Figure 3(*c*)]^45^.

Formation of estrogen-DNA adducts has also been associated with cancer in men^46-48^, and the same adduct ratio can be used as a biomarker of risk. Urine samples from men with and without prostate cancer have been analyzed by UPLC-MS/MS^46,47^. In an initial study, diagnosis with prostate cancer was associated with significantly higher levels of the depurinating adduct 4-OHE_1_(E_2_)-1-N3Ade^46^. In a subsequent, larger study, the estrogen-DNA adduct ratio was significantly higher in men with prostate cancer than in controls [p<0.001, Figure 3(*d*)]^47^. These results suggest that formation of estrogen-DNA adducts plays a critical role in the etiology of prostate cancer.

In a similar small study of men diagnosed with non-Hodgkin lymphoma (NHL] plus healthy controls, the adduct ratio was significantly higher in men with NHL compared to controls [p<0.0007, Figure 3(*e*)]^48^. We think that investigation of other prevalent types of cancer will demonstrate that they, too, are initiated by formation of estrogen-DNA adducts. These cancers include brain, colon, endometrium, kidney, leukemia, lung of non-smokers, melanoma, myeloma, pancreas and testis.

In summary, the ratio of estrogen-DNA adducts to estrogen metabolites and conjugates was significantly higher in cases compared to controls in all five types of cancer studied: breast, ovarian, thyroid and prostate cancers, plus NHL. The high adduct ratios in women at high risk for breast cancer and the association of SNPs in CYP1B1 and COMT with increased odds of ovarian cancer provide particularly strong evidence for a critical role of estrogen-DNA adducts in the etiology of these cancers.

By using sensitivity and specificity curves for the ratio levels, an initial cut-point of 77 for breast cancer^43^, 43 for ovarian cancer^44^ and 30 for thyroid cancer^45^ was determined. This suggests that DNA adduct ratios above 77 indicate high risk for cancer, while ratios below 30 indicate low risk, while ratios of 30-77 are indeterminate. Additional studies with more subjects and other types of cancer will enable refinement of this potential biomarker of cancer risk.

## Prevention of cancer

When estrogen metabolism is unbalanced, the level of catechol estrogen quinones increases and then more depurinating estrogen-DNA adducts are formed. This can be inhibited by balancing estrogen metabolism through the use of specific dietary supplements such as *N*-acetylcysteine (NAC) and resveratrol (Res), These two compounds are particularly effective in preventing the formation of estrogen-DNA adducts because they inhibit formation of catechol estrogen quinones and/or their reaction with DNA^49^.

NAC has very low toxicity, but has multiple anticarcinogenic properties^50,51^ and can generate the cellular scavenger GSH. NAC reacts efficiently with the electrophilic E_1_(E_2_)-3,4-Q^49^, preventing them from forming adducts with DNA. By reducing catechol estrogen semiquinones to catechol estrogens (Figure 2)^52^ and/or reacting with E_1_(E_2_)-3,4-Q, NAC prevents malignant transformation of human MCF-10F cells^53^ and mouse E6 mammary cells^39^ treated with 4-0HE_2_.

**Figure 4:**
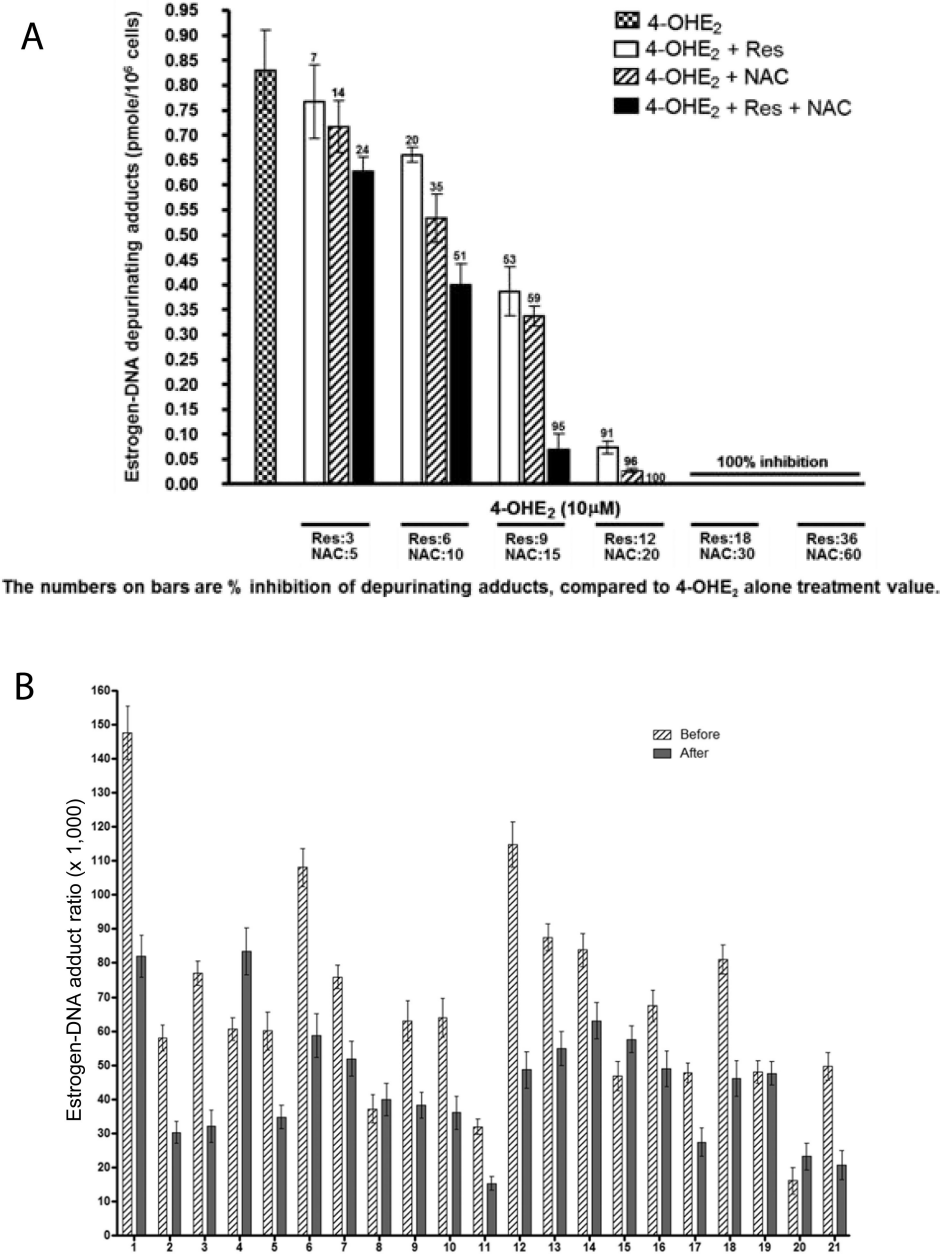
(*a*) Effects of NAC, Res, or NAC plus Res on the formation of depurinating estrogen-DNA adducts in MCF-10F cells treated with 4-OHE_2_. The number above each bar indicates the percent inhibition compared to treatment with only 4-OHE_2_^59^. (*b*) Estrogen-DNA adduct ratios in women before and after following the Healthy Breast Protocol for three months^60^.

Both NAC and Res can cross the blood-brain barrier^50,51,54,55^. Res has chemopreventive effects^54,55^, can modulate CYP1B1^38,56^, induce quinone reductase^38,57^ and reduce catechol estrogen semiquinones to catechol estrogens^38^. Res inhibits formation of estrogen-DNA adducts in MCF-10F cells treated with 4-OHE_2_^38,58^. When MCF-10F cells were treated with 4-OHE_2_ and NAC, Res or NAC plus Res, the compounds inhibited formation of depurinating estrogen-DNA adducts in an additive manner [p<0.0001, Figure 4(*a*)]^59^.

The effects of NAC and Res were studied as part of a Healthy Breast Protocol for women^60^. Healthy women, never diagnosed with cancer, followed the Healthy Breast Protocol daily for three months and provided a spot urine sample immediately before and after the treatment. The urine samples were analyzed for estrogen metabolites, estrogen conjugates and depurinating estrogen-DNA adducts by using UPLC-MS/MS, and the adduct ratio was calculated for each sample [Figure 4(*b*)]. Among the 21 participants, 16 showed lower adduct ratios after treatment, four showed no change and one had a higher ratio. The average decrease in adduct ratio after treatment with the Healthy Breast Protocol was statistically significant [p<0.03]^60^. These results indicate that a treatment protocol with NAC and Res can reduce formation of depurinating estrogen-DNA adducts in people.

In summary, NAC and Res have a variety of effects that can play a role in reducing formation of estrogen-DNA adducts, thus reducing the risk of developing cancer.

## Conclusions

Imbalanced estrogen metabolism can lead to excessive formation of carcinogenic catechol estrogen-3,4-quinones. Reaction of these quinones with DNA predominantly leads to depurinating estrogen-DNA adducts that can generate mutations to initiate many prevalent types of human cancer. These adducts can serve as biomarkers for risk of developing cancer.

Since formation of depurinating estrogen-DNA adducts is a critical event in cancer initiation, reducing their formation can reduce the risk of developing cancer. *N*-acetylcysteine and resveratrol impede formation of these adducts through complementary mechanisms, suggesting a widely applicable approach to cancer prevention. Since preventing cancer-leading mutations would stop the development of cancer, it is not necessary to know which mutations lead to which types of cancer. This is one of the reasons why preventing formation of estrogen-DNA adducts can be such a powerful cancer prevention tool.
